# Blocking circ_UBR4 suppressed proliferation, migration, and cell cycle progression of human vascular smooth muscle cells in atherosclerosis

**DOI:** 10.1515/biol-2021-0044

**Published:** 2021-04-27

**Authors:** Ying Zhang, Cheng Zhang, Zongwei Chen, Meilan Wang

**Affiliations:** Department of Cardiology, Zhongshan Affiliated Hospital, Dalian University, No. 6, Zhonshan Road, Dalian, 116001, Liaoning, China

**Keywords:** circ_UBR4, miR-107, ROCK1, atherosclerosis, VSMCs

## Abstract

The circ_UBR4 (hsa_circ_0010283) is a novel abnormally overexpressed circRNA in oxidized low-density lipoprotein (ox-LDL)-induced model of atherosclerosis (AS) in human vascular smooth muscle cells (VSMCs). However, its role in the dysfunction of VSMCs remains to be further investigated. Here, we attempted to explore its role in ox-LDL-induced excessive proliferation and migration in VSMCs by regulating Rho/Rho-associated coiled-coil containing kinase 1 (ROCK1), a therapeutic target of AS. Expression of circ_UBR4 and ROCK1 was upregulated, whereas miR-107 was downregulated in human AS serum and ox-LDL-induced VSMCs. Depletion of circ_UBR4 arrested cell cycle, suppressed cell viability, colony-forming ability, and migration ability, and depressed expression of proliferating cell nuclear antigen and matrix metalloproteinase 2 in VSMCs in spite of the opposite effects of ox-LDL. Notably, ROCK1 upregulation mediated by plasmid transfection or miR-107 deletion could counteract the suppressive role of circ_UBR4 knockdown in ox-LDL-induced VSMCs proliferation, migration, and cell cycle progression. In mechanism, miR-107 was identified as a target of circ_UBR4 to mediate the regulatory effect of circ_UBR4 on ROCK1. circ_UBR4 might be a contributor in human AS partially by regulating VSMCs’ cell proliferation, migration, and cell cycle progression via circ_UBR4/miR-107/ROCK1 pathway.

## 1 Introduction

Atherosclerosis (AS), a common cause of cardiovascular and cerebrovascular diseases, is a chronic inflammatory disease in blood vessels [1]. AS is initially distinguished by the formation of lipid-rich plaques in the wall of medium-to-large sized arteries [2]. Because AS is a progressive process, circulating oxidized lipids accumulate in inner layers of artery walls and then internalized by resident macrophages, eventually leading to the establishment of atheromatic plaque core [3]. Endothelial dysfunction followed by inflammation of the vessel wall leads to atherosclerotic lesion formation [4]. The excessive migration, proliferation, and inflammation of vascular smooth muscle cells (VSMCs), one essential component of blood vessels, are important pathological features after vascular injury in AS [5,6].

Circular RNAs (circRNAs) are endogenous and stable transcripts formed by back-splicing events. Emerging evidence demonstrates that circRNAs have been implicated in multiple cardiovascular diseases (CVDs) including AS [7], even supposed as potential theranostics [8]. VSMCs have long been considered as one key cellular target in preventing AS [9], and circRNA biology in VSMCs has also been extensively overviewed [10]. The circ_UBR4 (hsa_circ_0010283), located in the chromosome 1 and derived from exons 45 to 66 of UBR4, is one novel highly expressed circRNA in abnormal lipid metabolism in VSMCs stimulated by oxidized low-density lipoprotein (ox-LDL) [11]. ox-LDL is a biomarker of CVDs [12], and ox-LDL force could mimic inflammatory environment and lipid deposition in arterial wall cells. However, the specific molecular functions and biological effects of circ_UBR4 remain to be further defined.

circRNAs and competing endogenous RNAs (ceRNAs) are described as the “professional” microRNAs (miRNAs) “inhibitors/sponges” to regulate their cellular concentrations [13,14]. As miRNA-target interaction is strongly concentration dependent, circRNAs could depress the expression of functional genes and then participate in diverse cell progressions [15]. Rho-associated coiled-coil containing kinase 1 (ROCK1) is one effector of small GTP-binding protein Rho, and Rho/ROCK1 pathway has been developed as therapeutic agent for CVDs [16], as well as therapeutic targets for statins in AS [17]. The facilitating role of ROCK1 has been well documented in dysfunction of VSMCs and vascular endothelial cells in AS models *in vitro* and *in vivo* [18,19,20]. Yet, miRNA regulatory mechanism on ROCK1 in AS is poorly illustrated, especially the interplay among circRNAs, miRNAs, and ROCK1.

During atherogenesis, big epigenetic changes in different noncoding RNAs, especially miRNAs, are discovered in VSMCs, vascular endothelial cells, and macrophages [21]. miRNA (miR)-107, belonging to miR-15/107 family [22], has been found to be implicated in the pathogenesis of some diseases [23], including in vascular endothelial cells dysfunctions [24,25,26]; however, its role in VSMCs remains largely uncovered. In this study, we aimed to investigate the expression and role of circ_UBR4 in human VSMCs induced by ox-LDL; furthermore, the relationship and reciprocal role of circ_UBR4, ROCK1, and miR-107 in proliferation, migration, and cell cycle progression of this model cells were validated.

## 2 Materials and methods

### 2.1 Clinical serum samples

A group of 41 AS patients and 41 normal control people were recruited from Zhongshan Affiliated Hospital, Dalian University. The blood samples were harvested, and the serum was extracted by centrifugation at 3,000 g for 10 min. The control volunteers were without cancer, infection, or autoimmune diseases. No significant differences had been found in age and sex among patients and healthy volunteers, and more clinicopathological data of all participators are presented in Table 1.

**Table 1:** Clinicopathological data of AS patients and healthy volunteers

Clinical characteristics	Groups		*P*
	Normal (*N* = 41)	AS (*N* = 41)	
Gender, % (male)	63.41	68.29	>0.05
Age (years)	53 ± 7	55 ± 11	>0.05
Hypertension, % (*n*)	43.91 (18)	46.34 (19)	>0.05
Diabetes, % (*n*)	26.83 (11)	21.95 (9)	>0.05
Mean heart rate (bpm)	75.34 ± 11.63	78.26 ± 2.67	>0.05
Current cigarette user, % (*n*)	55.11	62.3	>0.05
Current alcohol user, % (*n*)	31.2	33.4	>0.05


**Informed consent:** Informed consent has been obtained from all individuals included in this study.
**Ethical approval:** The research related to human use has been complied with all the relevant national regulations and institutional policies and in accordance with the tenets of the Helsinki Declaration, and has been approved by the Ethics Committee of Zhongshan Affiliated Hospital, Dalian University.

### 2.2 Cell culture

Human VSMCs (BNCC340087) were obtained from BeNa Culture Collection (Beijing, China) and cultivated in Dulbecco’s Modified Eagle’s Medium, high glucose (M22650; R&D SYSTEMS, Shanghai, China) equipped with 10% fetal bovine serum (S11150H; R&D SYSTEMS). For AS model establishment *in vitro*, VSMCs at 80% confluence were administrated with 20, 40, 80, or 100 μg/mL ox-LDL (YB-002; Yiyuanbiotech, Guangzhou, China) for 24 h. Control cells were VSMCs without ox-LDL treatment. All cells were incubated in a humidified 5% CO_2_ incubator at 37°C.

### 2.3 Reverse transcriptase quantitative PCR (RT-qPCR)

Total RNA was isolated in TRIzol LS (for serum; Invitrogen, Carlsbad, CA, USA) and TRIzol (for cells; Invitrogen) according to its manufacturer protocol. The RNA samples were subjected to RT-qPCR with SuperScript First-strand Synthesis System (Invitrogen) with gDNA remover for cDNA synthesis and SYBR Green qPCR Master Mix (Invitrogen) for cDNA amplification. The special qPCR primers for circ_UBR4, UBR4, miR-107, ROCK1, glyceraldehyde-phosphate dehydrogenase (GAPDH), and U6 were provided by GENEWIZ (Beijing, China), and the sequence of primers is presented in Table 2. Cycle threshold (Ct) values were determined on 7500 real-time PCR System (Applied Biosystems, Foster City, MA, USA), and the condition of running RT-qPCR is presented in Table 3. Relative expression was calculated using the 2^−ΔΔCt^ method. GAPDH (for circRNA and mRNA) and U6 (for miRNA) were used as endogenous controls.

**Table 2:** The primers of RT-qPCR

Name/length	Sequence (5′–3′)	Specificity (≤length)
circ_UBR4 (112nt)	5′-ACACTCATCAGCCTGTTCCA-3′ forward and 5′-CCCTGTAGTTTGCTGGACAC-3′ reverse	Yes https://www.ncbi.nlm.nih.gov/tools/primer-blast/circ_UBR4 FR
UBR4 (118nt)	TTCCCCTCGAAGCAACACTC forward	Yes https://www.ncbi.nlm.nih.gov/tools/primer-blast/UBR4 FR
	CTGGCTTTCCTGACGGATGT reverse	
miR-107 (74nt)	5′-CAGCATTGTACAGGGCT-3′ forward and 5′-GAACATGTCTGCGTATCTC-3′ reverse	Yes https://www.ncbi.nlm.nih.gov/tools/primer-blast/miR-107 FR
ROCK1 (136nt)	AAGAGGGCATTGTCACAGCA forward	Yes https://www.ncbi.nlm.nih.gov/tools/primer-blast/ROCK1 FR
	AGCATCCAATCCATCCAGCA reverse	
GAPDH (101nt)	ACAACTTTGGTATCGTGGAAGG forward	Yes https://www.ncbi.nlm.nih.gov/tools/primer-blast/GAPDH FR
	GCCATCACGCCACAGTTTC reverse	
U6 (87nt)	5′-CGCTTCGGCAGCACATATAC-3′ forward and 5′-TTCACGAATTTGCGTGTCATC-3′ reverse	Yes https://www.ncbi.nlm.nih.gov/tools/primer-blast/U6 FR

**Table 3:** The conditions of running RT-qPCR

Procedure	Temperature (°C)	Time (s)
Pre-denaturation	95	30
Denaturation (for 40 cycles)	95	5
Annealing/extension (for 40 cycles)	62	20

### 2.4 Ribonuclease R (RNase R) and actinomycin D (ActD) treatment

RNase R is an exoribonuclease that degraded linear RNA but left circular transcripts intact [27]. The extracted RNA in 100 μg/mL ox-LDL-treated VSMCs was treated with RNase R (Geneseed, Guangzhou, China) with a ratio of 3 U enzyme/μg RNA for 30 min at 37°C or treated with the 1× Reaction Buffer (as Mock group). Ethanol precipitation was carried out to remove the enzyme and salts. After that, RT-qPCR was used to examine circ_UBR4 and UBR4 expression. ActD was used to block transcription, and 100 μg/mL ox-LDL-treated VSMCs in 24-well plate were added to 2 μg/mL ActD (Amyjet, Wuhan, China) for 0, 4, 8, 16, and 24 h before total RNA isolation and RT-qPCR for the detection of circ_UBR4 and UBR4 expression.

### 2.5 Cell transfection

VSMCs in 60% confluence were performed for cell transfection with the aid of Lipofectamine 3000 (Invitrogen) following the manufacturer’s protocols. The nucleotides included siRNA targeting circ_UBR4 (si-circ_UBR4), miR-107 mimic (miR-107), miR-107 inhibitor (anti-miR-107), and the negative controls (si-NC, miR-NC, and anti-miR-NC), as well as pcDNA plasmid (pcDNA) and pcDNA-ROCK1 plasmid (ROCK1). Post-transfection for 24 h, VSMCs were subjected to 100 μg/mL ox-LDL treatment for 24 h. The sequence of oligonucleotides was listed, including si-circ_UBR4, 5′-CAGCCTGTTCCAATGGTGGCA-3′; miR-107, 5′-AGCAGCAUUGUACAGGGCUAUCA-3′; anti-miR-107, 5′-TGATAGCCCTGTACAATGCTGCT-3′; si-NC, 5′-UUCUCCGAACGUGUCACGUTT-3′; miR-NC, 5′-GGUUCGUACGUACACUGUUCA-3′; anti-miR-NC, 5′-CAGUACUUUUGUGUAGUACAA-3′.

### 2.6 Flow cytometry (FCM)

Cell cycle distribution of VSMCs in control group and ox-LDL group was determined using propidium iodide (PI; Sigma-Aldrich, St. Louis, MO, USA) staining and FCM analysis. VSMCs were collected and prepared before testing. Briefly, 1 × 10^6^ cells were digested with trypsin free from EDTA and then fixed in 70% ethanol overnight at 4°C. After RNase A treatment (100 μg/mL, 1 h, 37°C), the cells were stained with 50 μg/mL PI at room temperature for 20 min. Promptly, the DNA contents of stained cells were analyzed on FlowJo software 7.6 (Tree Star, San Carlos, CA, USA).

### 2.7 Colony formation assay and cell counting kit (CCK)-8 assay

Control VSMCs and ox-LDL-treated VSMCs were seeded into 6-well plate at a density of 300 cells/well, and these cells were cultured in complete medium for another 14 days. Fresh medium was replaced per 3 days. Then, cells were fixed with 70% ethanol for 30 min and stained with 0.2% crystal violet for 30 min at the room temperature. Images of the colonies formed were captured by Quantity One (Bio-Rad, Hercules, CA, USA).

Cell viability of VSMCs before and after ox-LDL treatment was measured by CCK-8 assay; 20 μL of CCK-8 solution (Sigma-Aldrich) was added to the medium of each well, and five repeated wells were set up in each group. After incubation for 2 h at 37°C, the optical density (OD) value at 570 nm was measured using an automatic multi-well spectrophotometer (Bio-Rad).

### 2.8 Transwell assay

Cell migration ability was evaluated by transwell assay with transwell chamber (6.5 μm pore; Corning, NY, USA). Control VSMCs and ox-LDL-treated VSMCs were re-suspended in serum-free medium at a density of 1 × 10^4^ cells/chamber, followed by transferring in the top chamber. Another 400 μL of complete medium was placed into the lower chamber. Transwell chambers were maintained at 37°C for 48 h. The migrated cells on the basolateral chamber were fixed with 70% ethanol for 30 min and stained with 0.2% crystal violet for 30 min at room temperature. The stained cells were captured under an inverted microscope (Olympus, Tokyo, Japan).

### 2.9 Western blotting

Total cellular proteins of VSMCs were lysed in RIPA buffer supplemented with protease inhibitors, phosphatase inhibitors, and phenylmethanesulfonyl fluoride (PMSF). With centrifugation at 12,000 *g* for 20 min at 4°C, protein samples were harvested. The concentration of obtained proteins was quantified using Bradford reagent (Sigma-Aldrich), and 20 μg proteins were separated, transferred, and exposed following the standard procedures [28]. The primary antibodies against ROCK1 (ab45171, 1:10,000), proliferating cell nuclear antigen (PCNA; ab18197, 1:1,000), matrix metalloproteinase 2 (MMP2; ab97779, 1:2,500), and GAPDH (ab9485, 1:2,500) were from Abcam (Cambridge, UK), as well as HRP-conjugated secondary antibody against rabbit (ab205718, 1:50,000). Staining intensity of the bands was detected on Integrated chemiluminescence apparatus (ChemiScope 5300 Pro, Clinx, Shanghai, China). GAPDH was the internal control.

### 2.10 Dual-luciferase reporter assay

Artificially synthesized fragments of circ_UBR4 and ROCK1 3′ untranslated region (3′UTR) containing miR-107-binding sites were, respectively, inserted into the luciferase report vector pmirGLO (Promega, Madison, WI, USA), named as circ_UBR4-wt and ROCK1 3′UTR-wt. A mutation of these sites was performed and inserted into pmirGLO (Promega) as well, named as circ_UBR4-mut and ROCK1 3′UTR-mut. VSMCs seeded in 24-well plate were co-transfected with pmirGLO vectors carrying circ_UBR4-wt, ROCK1 3′UTR-wt, circ_UBR4-mut, or ROCK1 3′UTR-mut together with miR-107 or miR-NC. The transfection was performed in triplicate. Post transfection for 48 h, the Firefly luciferase activity was examined by Dual Luciferase Assay System (Promega) and normalized to the Renilla luciferase activity.

### 2.11 Pearson’s correlation analysis

The correlation among circ_UBR4, miR-107, and ROCK1 mRNA levels in serum samples from AS patients (*n* = 41) was identified using Pearson’s correlation analysis. The correlation coefficient was greater than 0.8.

### 2.12 Statistical analysis

All data were analyzed with software GraphPad Prism 6 (GraphPad Software, La Jolla, CA, USA), and data were presented as mean ± standard deviation for at least three times from biological level. Statistical analyses were performed using unpaired Student’s *t*-test or one-way analysis of variance (ANOVA). Tukey’s post hoc analysis was performed after ANOVA. *P* < 0.05 was deemed as significantly different.

## 3 Results

### 3.1 circ_UBR4 was upregulated in human AS serum and ox-LDL-induced human VSMCs with a stable structure

**Figure 1:**
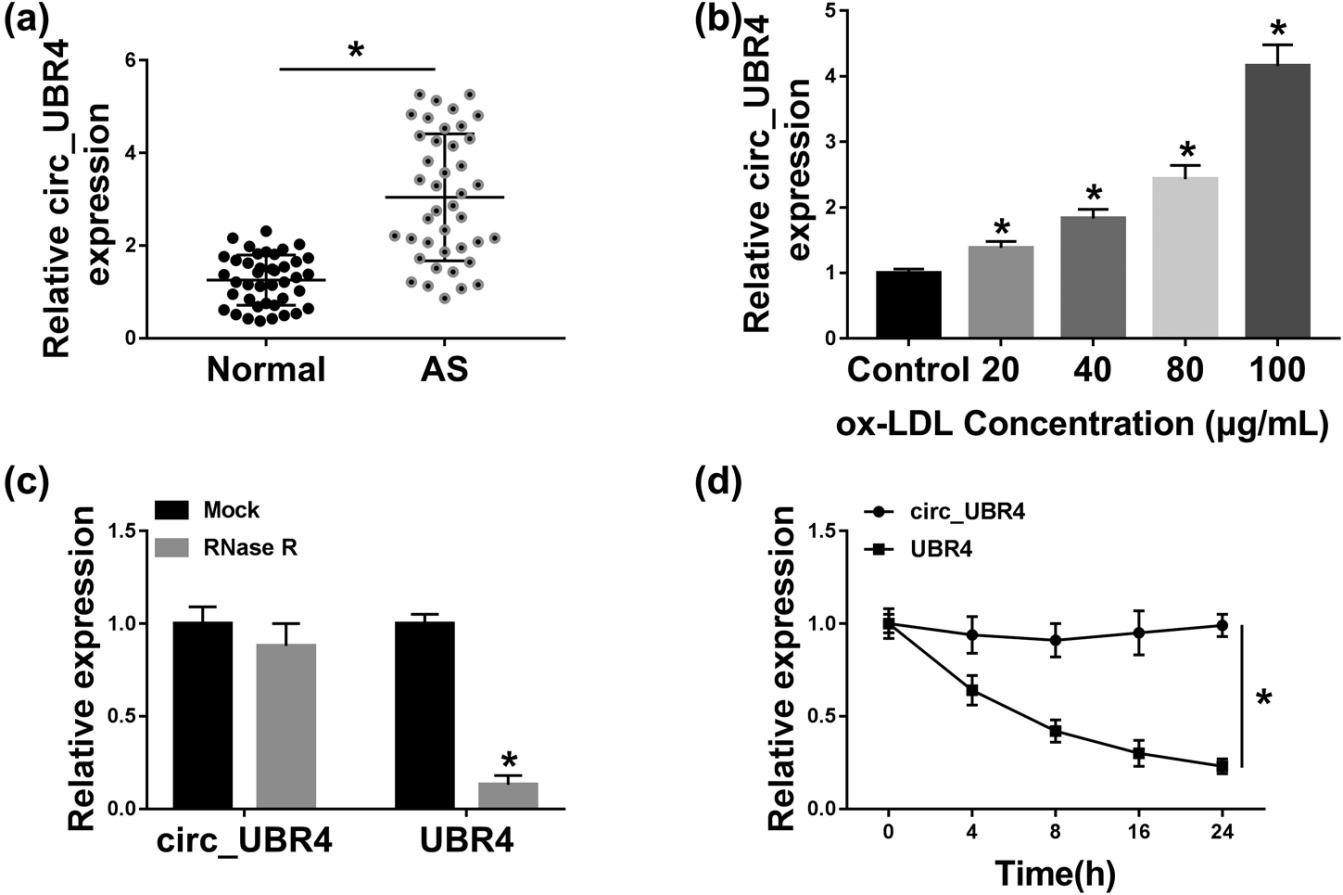
Expression of circ_UBR4 (hsa_circ_0010283) was upregulated in human AS serum and ox-LDL-induced VSMCs. RT-qPCR detected circ_UBR4 expression level in (a) serum samples from AS patients (AS; *n* = 41) and normal control people (normal; *n* = 41), and (b) human VSMCs treated with different concentrations of ox-LDL for 24 h. (c and d) RT-qPCR measured levels of circ_UBR4 and its host gene UBR4 in (c) extracted RNA treated with RNase R or with Mock, and (d) 100 μg/mL ox-LDL-treated VSMCs treated with ActD. *P < 0.05.

To screen circ_UBR4 expression in AS patients, serum samples were collected. The level of circ_UBR4 in human AS serum was 3.1-fold of that in normal people (Figure 1a). *In vitro*, human VSMCs were disposed to ox-LDL to stimuli AS model, and circ_UBR4 level was increased in ox-LDL-treated VSMCs (Figure 1b). Besides, compared with its host gene UBR4, circ_UBR4 expression was unaffected with RNase R and ActD treatment (Figure 1c and d). These results suggested a potential role of circ_UBR4 underlying the pathogenesis of AS, especially in VSMCs.

### 3.2 The blockage of circ_UBR4 restrained proliferation, migration, and cell cycle progression of ox-LDL-induced human VSMCs

**Figure 2:**
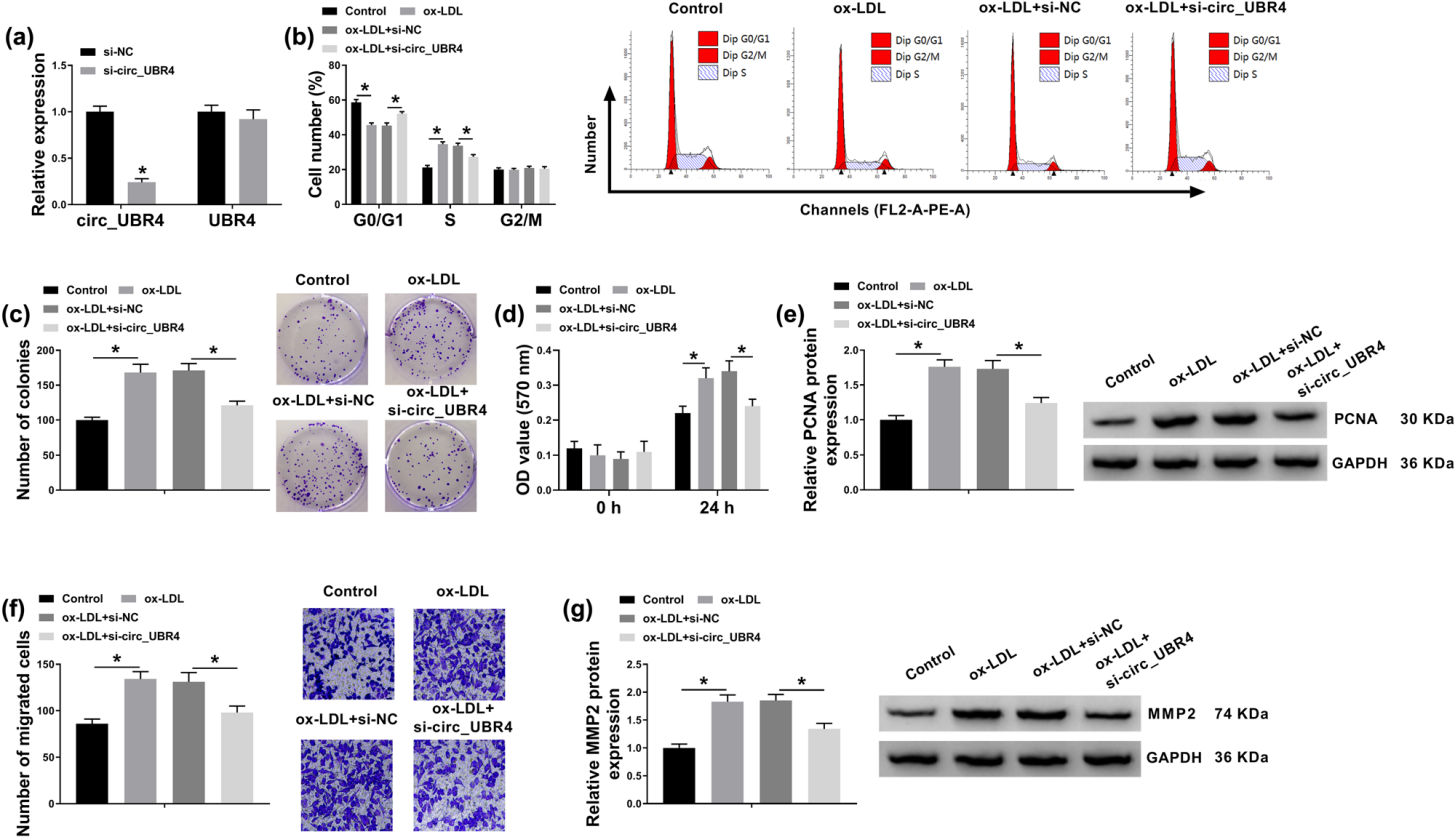
Suppressive role of circ_UBR4 depletion in cell cycle progression, proliferation, and migration of ox-LDL-induced human VSMCs. (a) RT-qPCR detected circ_UBR4 and UBR4 expression levels in VSMCs transfected with siRNA targeting circ_UBR4 (si-circ_UBR4), normalized to its negative control (si-NC) transfection. (b–g) VSMCs were co-treated with siRNA transfection for 24 h and ox-LDL (100 μg/mL) treatment for another 24 h. (b) FCM analyzed cell distributions in different cell cycle phases (G0/G1, S, and G2/M) by counting cell number. (c) Colony formation assay measured colony-forming ability by determining number of colonies. (d) CCK-8 assay monitored cell viability by analyzing OD value at 570 nm. (e) Western blotting detected expression of proliferation marker, PCNA. (f) Transwell assay evaluated cell migration ability by counting number of migrated cells. (g) Western blotting detected expression of migration marker, matrix MMP2. *P < 0.05.

To investigate the cellular function of circ_UBR4 in human VSMCs, circ_UBR4 was artificially silenced using siRNA transfection, and RT-qPCR identified a high knockdown efficiency of si-circ_UBR4 in VSMCs (Figure 2a). Loss-of-function experiments were carried out in ox-LDL-treated VSMCs with siRNA transfection. ox-LDL treatment induced higher cell distribution in S-phase and lower cell distribution in G0/G1 phase (Figure 2b), whereas these cell distributions were reversed with si-circ_UBR4 pre-transfection. Colony-forming ability of VSMCs was facilitated in response to ox-LDL treatment, which was attenuated by introducing si-circ_UBR4 (Figure 2c). CCK-8 assay showed cell viability promotion in ox-LDL-treated VSMCs (Figure 2d), accompanied with elevated expression of PCNA (proliferation marker) (Figure 2e). Moreover, si-circ_UBR4-mediated circ_UBR4 downregulation led to diminishment of cell viability and PCNA expression in ox-LDL-challenged VSMCs (Figure 2d and e). Transwell migration ability of VSMCs along with expression of MMP2 (migration marker) was promoted under ox-LDL stress, and this promotion was cancelled with pre-transfection of si-circ_UBR4 (Figure 2f and g). Above outcomes indicated a suppressive effect of circ_UBR4 blockage on ox-LDL-induced excessive proliferation, migration, and cell cycle progression of human VSMCs *in vitro*.

### 3.3 ROCK1 overexpression counteracted the suppressive role of circ_UBR4 depletion in ox-LDL-induced human VSMCs

**Figure 3:**
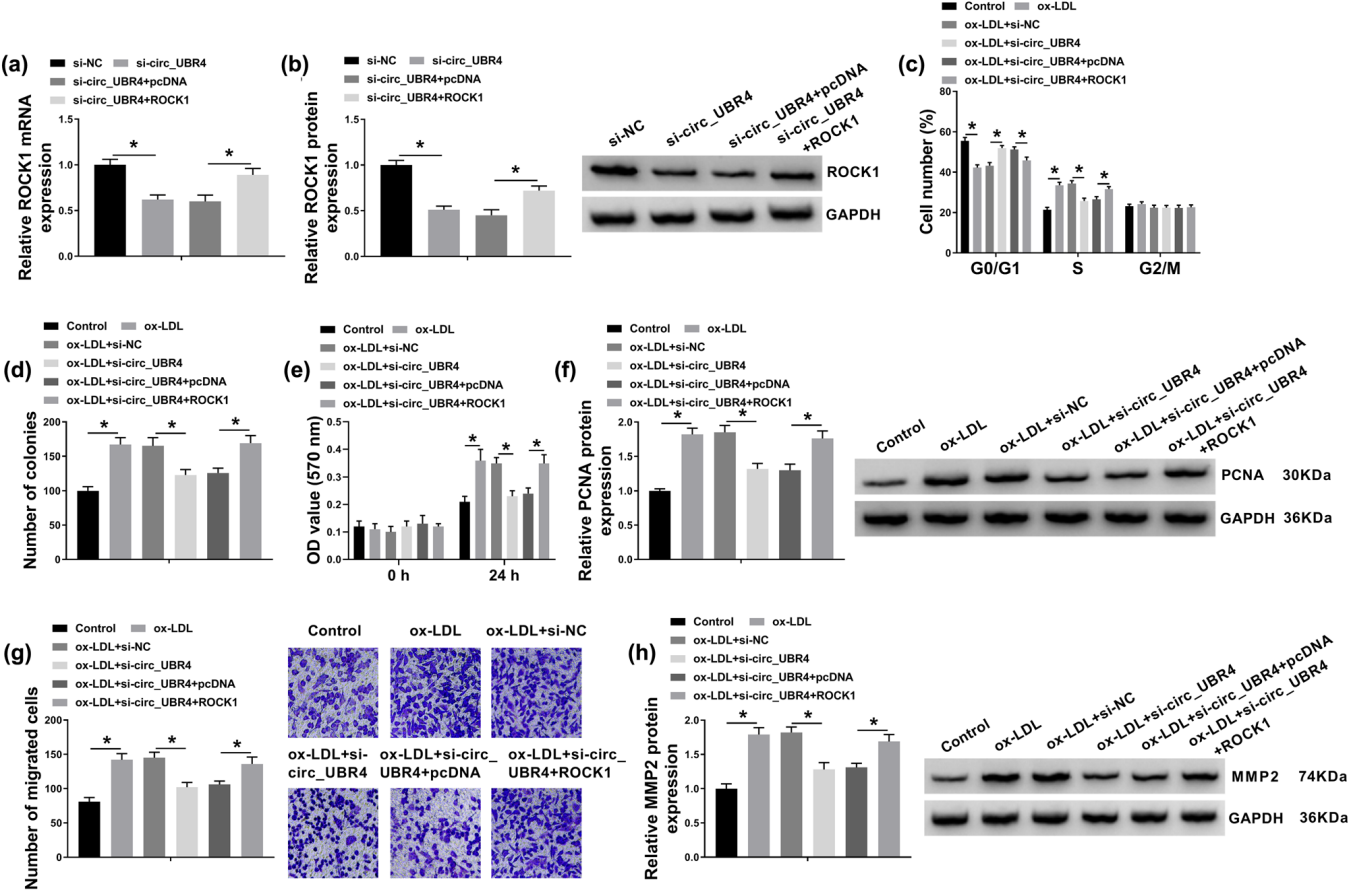
Rho/Rho-associated coiled-coil containing kinase 1 (ROCK1) counteracted the role of circ_UBR4 depletion in ox-LDL-induced human VSMCs. (a and b) RT-qPCR and western blotting examined ROCK mRNA expression level and protein expression level in VSMCs transfected with si-NC, si-circ_UBR4, si-circ_UBR4 along with pcDNA plasmid (pcDNA), si-circ_UBR4 along with pcDNA-ROCK1 plasmid (ROCK1). (c–h) VSMCs were co-treated with above transfections for 24 h and ox-LDL (100 μg/mL) treatment for another 24 h. (c) FCM analyzed cell numbers in G0/G1, S, and G2/M phases. (d) Colony formation assay determined number of colonies. (e) CCK-8 assay monitored OD value at 570 nm. (f) Western blotting detected PCNA protein expression level. (g) Transwell assay evaluated number of migrated cells. (h) Western blotting detected MMP2 protein expression level. *P < 0.05.

ROCK1 plasmid transfection restored the expression of ROCK1 on both mRNA level and protein level in VSMCs transfected with si-circ_UBR4 (Figure 3a and b). Moreover, ROCK1 plasmid also abrogated the suppression of circ_UBR4 silence on cell cycle progression, as depicted by decreased G0/G1 cells and increased S-cells (Figure 3c). Colony number of ox-LDL-treated VSMCs was reduced by silencing circ_UBR4, and this reduction was mitigated with the presence of ROCK1 plasmid (Figure 3d). Under ox-LDL stimuli, si-circ_UBR4 lowered cell viability and PCNA expression of VSMCs, which was rescued because of co-administration of ROCK1 plasmid (Figure 3e and f). Anti-migration role of circ_UBR4 knockdown in ox-LDL-disposed VSMCs was augmented by restoring ROCK1 expression via plasmid transfection, as evidenced by increased transwell migrated cells and higher MMP2 protein level (Figure 3g and h). These findings demonstrated that ROCK1 upregulation could counteract the suppression of circ_UBR4 knockdown on ox-LDL-induced VSMCs excessive proliferation, migration, and cell cycle progression.

### 3.4 miR-107 directly interacted with both circ_UBR4 and ROCK1 in human VSMCs

**Figure 4:**
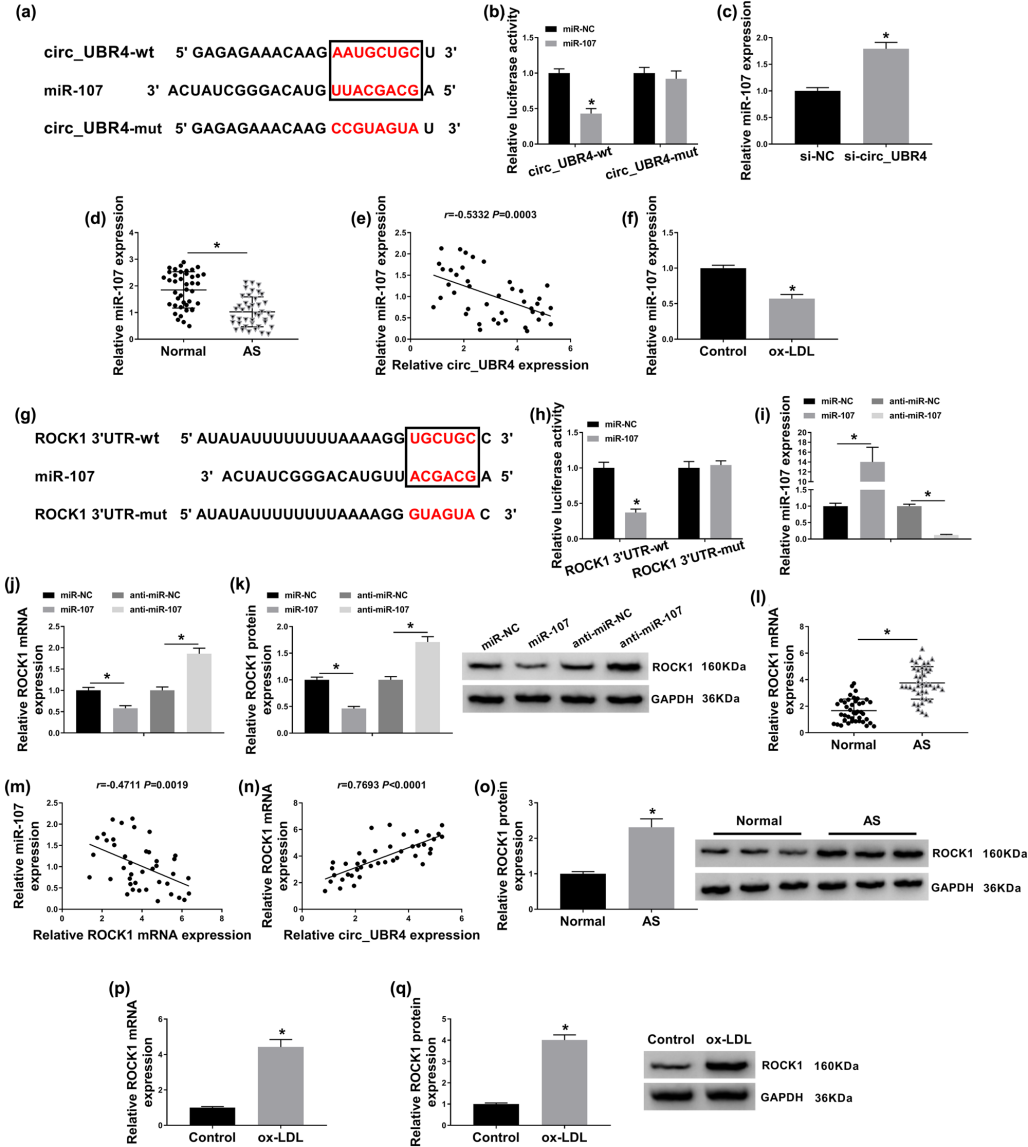
miRNA (miR)-107 directly interacted with circ_UBR4 and ROCK1. (a and g) Starbase3.0 software predicted the potential complementary sites of miR-107 on (a) wild type of circ_UBR4 (circ_UBR4-wt) and (g) wild type of ROCK1 in 3′untranslated region (ROCR1 3′UTR-wt). The red in black box was the binding sites, and the red out of black box was the mutated sites in the mutant type of circ_UBR4 (circ_UBR4-mut) and mutant type of ROCK1 (ROCK1 3′UTR-mut). (b and h) Dual-luciferase reporter assay measured luciferase activity of vector carrying (b) circ_UBR4-wt or circ_UBR4-mut, and (h) ROCR1-wt or ROCR1-mut in VSMCs transfected with miR-107 mimic (miR-107) or its negative control (miR-NC). (c and d) RT-qPCR detected miR-107 expression level in VSMCs transfected with si-NC or si-circ_UBR4, and serum samples in AS and normal groups. (e) Pearson’s correlation analysis determined the association between circ_UBR4 and miR-107 expression in human AS serum. (f) RT-qPCR detected miR-107 expression level in ox-LDL (100 μg/mL)-treated human VSMCs. (i–k) VSMCs were transfected with miR-NC, miR-107, miR-107-5p inhibitor (anti-miR-107), or the negative control (anti-miR-NC), and then (i and j) RT-qPCR measured miR-107 expression level and ROCK1 mRNA expression level, and (k) western blotting examined ROCK1 protein expression level. (l) RT-qPCR examined ROCK1 mRNA expression level in serum samples in AS and normal groups. (m and n) Pearson’s correlation analysis determined the association between ROCK1 mRNA and either miR-107 or circ_UBR4 expression in human AS serum. (o) Western blotting detected ROCK1 protein expression level in three groups of serum samples from AS patients and normal people. (p and q) RT-qPCR and western blotting measured ROCK1 expression in ox-LDL (100 μg/mL)-treated human VSMCs both on mRNA level and protein level. *P < 0.05.

With the in silico data on starbase3.0 software (http://starbase/circRNA&miRNA/=UBR4/=miR-107, http://starbase/mRNA&miRNA/=ROCK1/=miR-107), miR-107 was complementary to both circ_UBR4 and ROCK1. According to the predicted miR-107-binding site in circ_UBR4-wt and ROCK1 3′UTR-wt (Figure 4a and g), circ_UBR4-mut and ROCK1 3′UTR-mut were constructed. Dual-luciferase reporter assay revealed that luciferase activities of vectors containing circ_UBR4-wt and ROCK1 3′UTR-wt were declined in VSMCs transfected with miR-107 compared to miR-NC transfection (Figure 4b and h). These data hinted a target relationship between miR-107 and circ_UBR4 or ROCK1. In terms of miR-107 expression, its level was upregulated by si-circ_UBR4 transfection and miR-107 mimic transfection (Figure 4c and i), whereas downregulated by anti-miR-107 transfection (Figure 4i). In AS samples, miR-107 expression was lower in human AS serum and VSMCs under ox-LDL stress (Figure 4d and f). Moreover, miR-107 expression was negatively correlated with circ_UBR4 in AS patients’ serum (Figure 4e). On the contrary, ROCK1 expression on both mRNA level and protein level was inversely regulated by miR-107, as described by increased ROCK1 level in anti-miR-107-transfected VSMCs and decreased ROCK1 level in miR-107-transfected VSMCs (Figure 4j and k). In AS patients, ROCK1 expression level was higher in the serum samples comparing to normal people (Figure 4l and o), and its mRNA expression was negatively correlated with miR-107 and positively correlated with circ_UBR4 (Figure 4m and n). In addition, ROCK1 expression was highly expressed in ox-LDL-induced human VSMCs (Figure 4p and q). Taken together, miR-107 was abnormally expressed in AS probably through directly interacting with circ_UBR4 and ROCK1.

### 3.5 Silencing miR-107 abolished the suppressive role of circ_UBR4 depletion in ox-LDL-induced human VSMCs through upregulating ROCK1

**Figure 5:**
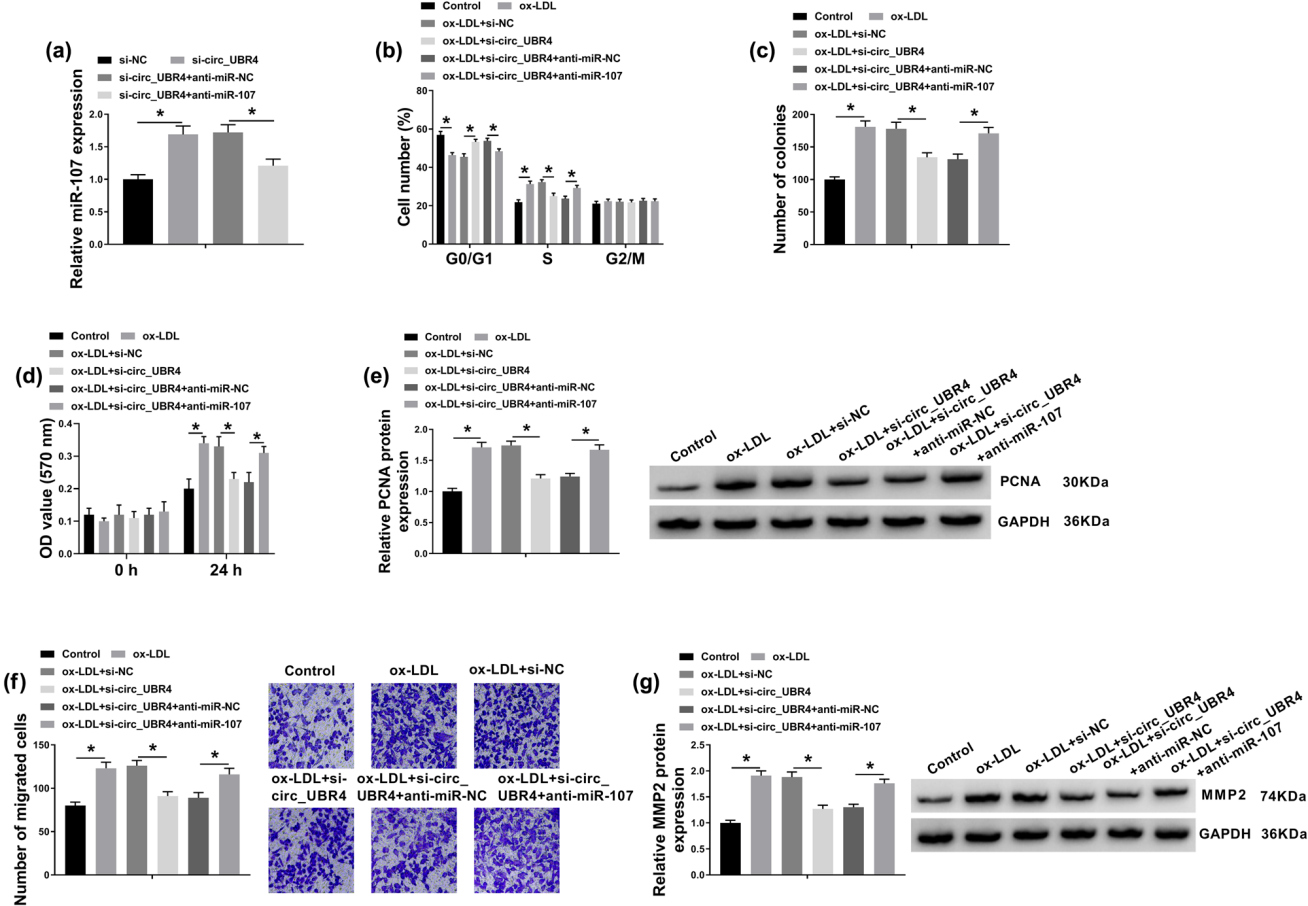
Silencing miR-107 abolished the role of circ_UBR4 depletion in ox-LDL-induced human VSMCs. (a) RT-qPCR examined miR-107 expression level in VSMCs transfected with si-NC alone, si-circ_UBR4 alone, or together with anti-NC or anti-miR-107. (b–g) VSMCs were co-treated with above transfections for 24 h and ox-LDL (100 μg/mL) treatment for another 24 h. (b) FCM analyzed cell numbers in G0/G1, S, and G2/M phases. (c) Colony formation assay determined number of colonies. (d) CCK-8 assay monitored OD value at 570 nm. (e) Western blotting detected PCNA protein expression level. (f) Transwell assay evaluated number of migrated cells. (g) Western blotting detected MMP2 protein expression level. *P < 0.05.

**Figure 6:**
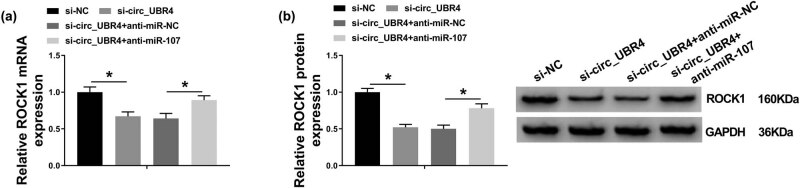
circ_UBR4 modulated ROCK1 expression in human VSMCs via miR-107 regulation. (a and b) RT-qPCR and western blotting measured ROCK1 expression levels in VSMCs transfected with si-NC alone, si-circ_UBR4 alone, or together with anti-NC or anti-miR-107. *P < 0.05.

The effect of miR-107 on the role of circ_UBR4 depletion in VSMCs in AS using rescue experiment was further explored. Blocking circ_UBR4 resulted in higher level of miR-107 in ox-LDL-treated human VSMCs, and anti-miR-107 presence could downregulate this upregulation (Figure 5a). Moreover, anti-miR-107 transfection caused a series of promoting effects on excessive cell progressions of ox-LDL-induced VSMCs with circ_UBR4 knockdown, including cell entrance to S phase (Figure 5b), colony-formation ability (Figure 5c), cell viability (Figure 5d), migration ability (Figure 5f), as well as PCNA and MMP2 expression (Figure 5e and g). Furthermore, in ox-LDL-induced VSMCs transfected with si-circ_UBR4, anti-miR-107 co-transfection not only suppressed miR-107 expression (Figure 5a) but also enhanced ROCK1 expression (Figure 6a and b). These outcomes illuminated that silencing miR-107 accompanied with ROCK1 upregulation could abolish the role of circ_UBR4 knockdown in human VSMCs under ox-LDL condition, suggesting a circ_UBR4/miR-107/ROCK1 regulatory axis in VSMCs underlying AS pathogenesis.

## 4 Discussion

Recently, differential expression and bioinformatics analysis of circRNA in AS tissues and cell models in VSMCs had been disclosed, both under platelet-derived growth factor (PDGF-BB) stress and ox-LDL pressure [11,29,30]. There were several circRNAs involved in the dysfunction of VSMCs and the pathogenesis of AS. For example, circ_CHFR depletion weakened ox-LDL-induced promotion of cell growth, migration, and inflammation in VSMCs through targeting miR-370/FOXO1/Cyclin D1 and miR-214-3p/Wnt3/β-catenin pathways [11,31]. Besides, VSMCs phenotypic differentiation, proliferation, apoptosis, and migration were regulated by circ_SATB2 and circ_RUSC2 via sponging miRNAs [32,33]. However, comprehensive analysis of the role of circRNAs in the dysfunction of VSMCs remained largely unclear. Thus, we selected a novel circRNA circ_UBR4 that was abnormally expressed in ox-LDL-stimulated VSMCs to further explore its role in VSMCs during AS.

Here, circ_UBR4 was upregulated in human AS serum and ox-LDL-induced VSMCs, which was consistent with the initial study [11,34]. Besides, the expression of circ_UBR4 was resistant to RNase R and ActD treatment, suggesting a stable structure of circ_UBR4 and its host gene UBR4. As for the function, ox-LDL-induced VSMCs’ excessive proliferation, colony-formation ability, migration, and cell cycle progression were attenuated by blocking circ_UBR4. Moreover, the suppressive role of circUBR4 knockdown in ox-LDL-induced proliferation, migration, and invasion in VSMCs had been previously demonstrated [34,35]. Molecularly, expression of PCNA and MMP2 was also depressed by blocking circ_UBR4 [35]; furthermore, circ_UBR4 modulated ox-LDL-evoked dysfunction of VSMCs through serving as a ceRNA for miRNAs, such as miR-133a-3p and miR-370-3p. In this study, we discovered that miR-107 was sponged by circUBR4 as well. These findings suggested a circ_UBR4-miR-107/133a-3p/370-3p-ROCK1/pregnancy-associated plasma protein A/high mobility group box 1 ceRNA network in modulating VSMCs proliferation and migration under ox-LDL pressure in AS.

In this present study, miR-107 was downregulated in human AS serum, and this finding was in favor of the previous outcomes [36]. Here, we also demonstrated the low level of miR-107 in ox-LDL-induced VSMCs, and this downregulation was also discovered in PDGF-BB-induced pulmonary arterial smooth muscle cells (PASMCs) [37]. In terms of cellular functions, we observed that miR-107 downregulation was associated with promotion of proliferation, migration, and cell cycle progression in ox-LDL-challenged human VSMCs in spite of the anti-AS role of circ_UBR4 deficiency. Our findings supported the notion that miR-107 blockage exhibited facilitating effect on proliferation and migration of PASMCs stimulated by PDGF-BB [37]. Moreover, Shen et al. [36] pointed out the link of miR-107 silence in VSMCs proliferation and invasion but also stated its involvement in proliferation and invasion of vascular endothelial cells under stimuli of lipopolysaccharide, a potentially important stimulator and AS risk factor. Besides, Gao et al. [24] indicated a pharmacological effect of miR-107 in coronary AS model in mouse, and miR-107 upregulation reduced blood lipid level and atherosclerotic index by suppressing vascular endothelial cells apoptosis, inflammation, and endoplasmic reticulum stress. Taken together, miR-107 might represent a new approach for pharmacological treatment of diseases including AS.

Except for KRT1 and NOR1 [24,37], ROCK1 was confirmed as a novel target gene of miR-107. ROCK1 was upregulated in the serum of AS patients, which was positively correlated with circ_UBR4 expression and negatively correlated with miR-107 expression. ROCK1 upregulation mediated the promotion of ox-LDL-induced excessive proliferation, migration, and cell cycle progression in VSMCs despite circ_UBR4 silencing exerting the opposite effects. Chen et al. [19] also noted similar facilitating role of ROCK1 in proliferation and migration of VSMCs induced by high glucose, also an *in vitro* model of AS. It was reported that ROCK1/2 suppression mediated by the inhibitor Y-27632 or siRNAs transfection could reduce expression of MMP2, belonging to MMP family that played a crucial role in the pathogenesis of CVDs [38]. Similar to this announcement, circ_UBR4 depletion accompanied ROCK1 downregulation to depress MMP2 expression. The suppressive effect of miR-107 on MMP2 expression might be first documented in this study. By the way, starbase software also predicted a potential binding between miR-107 and ROCK2, and ROCK2 was also involved in Rho/ROCKs pathway in regulating AS endothelial function and vascular inflammation [17]. This hypothesis should be further validated.

In terms of PCNA expression regulation, we noticed a suppression of miR-107, as well as a promotion of ROCK1 on PCNA expression in ox-LDL-induced VSMCs. Furthermore, PCNA together with cyclin D1 could be significantly decreased with miR-107 ectopic expression in lung cancer cells and colorectal cancer cells [39,40], suggesting that miR-107 was an important regulator of cell proliferation. MMP2, MMP9, PCNA, ROCK1, and ROCK2 were increased in a mouse model of AS, and the receptor antagonist of IL-8, playing a key part in VSMCs proliferation and migration, could suppress the development of AS by inhibiting ROCKs [41]. Intriguingly, Lu et al. [42] expounded that knockdown of PCNA inhibited ROCK1 expression in colon cancer HCT116 cells, whereas ROCK1 knockdown did not affect PCNA expression. This finding hinted that ROCK1 silence could be downregulating PCNA expression in a content-dependent manner, such as in VSMCs with high ox-LDL level.

Collectively, we demonstrated that silencing of circ_UBR4 could attenuate ox-LDL-induced excessive proliferation, migration, and cell cycle progression in human VSMCs via circ_UBR4/miR-107/ROCK1 ceRNA regulatory axis. This result might suggest circ_UBR4, miR-107, and ROCK1 as potential targets in therapeutic schemes of AS.
